# Health Promotion Interventions for Low-Income Californians Through Medi-Cal Managed Care Plans, 2012

**DOI:** 10.5888/pcd12.150269

**Published:** 2015-11-12

**Authors:** Desiree R. Backman, Neal D. Kohatsu, Brian M. Paciotti, Jennifer V. Byrne, Kenneth W. Kizer

**Affiliations:** Author Affiliations: Neal D. Kohatsu, Jennifer V. Byrne, California Department of Health Care Services, Sacramento, California; Brian M. Paciotti, Optum Data Management, Sacramento, California, and California Department of Health Care Services, Sacramento, California; Kenneth W. Kizer, Institute for Population Health Improvement, University of California Davis Health System, and University of California Davis, School of Medicine and Betty Irene Moore School of Nursing, Sacramento, California. Dr Backman is also affiliated with the Institute for Population Health Improvement, University of California Davis Health System, Sacramento, California.

## Abstract

**Introduction:**

Prevention is the most cost-effective approach to promote population health, yet little is known about the delivery of health promotion interventions in the nation’s largest Medicaid program, Medi-Cal. The purpose of this study was to inventory health promotion interventions delivered through Medi-Cal Managed Care Plans; identify attributes of the interventions that plans judged to have the greatest impact on their members; and determine the extent to which the plans refer members to community assistance programs and sponsor health-promoting community activities.

**Methods:**

The lead health educator from each managed care plan was asked to complete a 190-item online survey in January 2013; 20 of 21 managed care plans responded. Survey data on the health promotion interventions with the greatest impact were grouped according to intervention attributes and measures of effectiveness; quantitative data were analyzed using descriptive statistics.

**Results:**

Health promotion interventions judged to have the greatest impact on Medi-Cal members were delivered in various ways; educational materials, one-on-one education, and group classes were delivered most frequently. Behavior change, knowledge gain, and improved disease management were cited most often as measures of effectiveness. Across all interventions, median educational hours were limited (2.4 h), and median Medi-Cal member participation was low (265 members per intervention). Most interventions with greatest impact (120 of 137 [88%]) focused on tertiary prevention. There were mixed results in referring members to community assistance programs and investing in community activities.

**Conclusion:**

Managed care plans have many opportunities to more effectively deliver health promotion interventions. Establishing measurable, evidence-based, consensus standards for such programs could facilitate improved delivery of these services.

## Introduction

Four modifiable behaviors — lack of physical activity, poor nutrition, tobacco use, and alcohol abuse — are the major risk factors for a substantial portion of chronic disease morbidity and mortality (1–3). Preventing disease or its progression is the most cost-effective and practical way to promote population health (4). Studies, however, indicate variable delivery and suboptimal use of preventive services, particularly among low-income populations, including those enrolled in Medicaid and the Children’s Health Insurance Program (CHIP) (5–7).

The California Department of Health Care Services (DHCS) administers Medi-Cal, which is the largest Medicaid program in the United States. Eighty percent of the approximately 12 million members are enrolled in some form of Medi-Cal Managed Care Plan (MCP). The MCPs are contracted by DHCS to provide a range of medically necessary diagnostic and treatment services. These include clinical preventive services that are informed by the United States Preventive Services Task Force (USPSTF) and health promotion interventions (HPIs) designed to achieve behavior change and positive health outcomes (8–11). Despite contract requirements, little is known about the characteristics and effectiveness of HPIs delivered to Medi-Cal members.

As part of a departmentwide quality improvement initiative, the objectives of this study were to 1) inventory HPIs offered to Medi-Cal members in the areas of healthful eating, physical activity, alcohol and drug abuse prevention, breastfeeding, asthma management, and prevention and management of cardiovascular disease (CVD), type 2 diabetes, and overweight/obesity; 2) identify attributes of HPIs that MCPs judged to have the greatest impact on their Medi-Cal members; and 3) determine the extent to which MCPs referred Medi-Cal members to community assistance programs and sponsored health-promoting community activities. 

## Methods

This was a cross-sectional, descriptive study consisting of a survey in January 2013 of the lead health educator from each of the 21 contracted MCPs in the Medi-Cal system. Lead health educators were identified through a DHCS database and asked via email and telephone to complete the survey. In alignment with DHCS contract requirements, lead health educators have authority and oversight for the implementation of HPIs in their MCPs. Lead health educators also have primary responsibility for the full portfolio of health promotion programs delivered to their MCP populations, and those participating in this study had at least a master’s degree in public health, health education, or a related field.

### Survey development

A 190-item survey instrument was developed from October through December 2012. Each health behavior (healthful eating, physical activity, alcohol abuse prevention, and breastfeeding) was selected for the survey because it is an important contributor to reducing the risk of morbidity and mortality (1–3,12). Each disease (obesity, CVD, and type 2 diabetes) was included because it is a common, costly, and largely preventable chronic condition (13). Two other topics — drug abuse prevention and asthma management — were included because the MCP medical directors and health educators requested their inclusion during survey development. In addition, MCP contracts require the implementation of risk reduction, healthful lifestyle, health condition management, and self-care interventions covering all topics in this study as well as others, such as injury prevention, prevention of sexually transmitted diseases, prenatal care, and more (8–10).

Originally, we wanted to include smoking cessation in the assessment, but it was the subject of a survey in January 2012 (14), and plans were under way to conduct other smoking cessation–related surveys of MCPs. Thus, we excluded smoking cessation from the survey to enable the assessment of other health topics.

A draft survey was reviewed by 4 population health experts, who compared the questions with the study aims to establish face validity. The survey instrument was also reviewed by MCP medical directors, who suggested no major changes, and by the 21 lead health educators to assess ease of use, comprehension, readability, and inclusion of major health topics. Minor semantic and formatting changes were made on the basis of their feedback.

### Survey measures

The survey contained detailed instructions and a mix of open- and close-ended questions with multiple response categories. An “other, please specify” response was included in all questions with multiple responses (survey available upon request).

An initial set of questions asked respondents to describe the administrative oversight of their MCP health education system. The lead health educators were asked to provide their contact information, title, and credentials, and a brief description of their health education systems, and to specify the number of full-time equivalents dedicated to health education. The next section asked for general information about the MCPs’ interventions, including information on how content is delivered and how DHCS could complement their efforts. They were also asked to provide the name of each HPI offered to Medi-Cal members by behavior and disease category. The bulk of the survey, organized by behavior and disease, then focused on interventions that the MCPs judged to have the greatest positive impact on their Medi-Cal members (hereinafter referred to as greatest impact health promotion interventions [GIHPIs]). For each GIHPI from January through December 2012, the survey respondents were asked to state the intervention’s goals, describe the intervention, specify the number of hours of education provided and the number of Medi-Cal members reached, explain how effectiveness was assessed and measured, and document outcomes among participants. The intent of these questions was to capture data on what the MCPs thought were the most effective HPIs being delivered to Medi-Cal members during the study period. No information of this kind was previously available.

The final set of questions asked whether the MCPs referred Medi-Cal members to community assistance programs, such as California’s Supplemental Nutrition Assistance Program (known as CalFresh), the Special Supplemental Nutrition Program for Women, Infants, and Children (WIC), Temporary Assistance for Needy Families, housing or utility assistance, or education and job training programs, among others, to address some of the social determinants of health (15). Respondents were also asked whether their health plan sponsored activities to foster healthy communities, such as physical activity events, food pantries, farmers markets, or community gardens, among others.

Health educators were given 4 weeks to complete the survey, and 20 of the 21 MCPs submitted survey responses. Repeated attempts to obtain the one unanswered survey were unsuccessful.

### Statistical analysis

Online survey responses were downloaded into an Excel file and exported into SAS/STAT, version 9.3 (SAS Institute Inc). To protect privacy, all lead health educator and MCP names were removed from the data set before analysis and only aggregate analyses were conducted.

Data analysis, conducted from August 2013 through June 2014, consisted of 2 components. First, open-ended questions on program descriptions and measures of effectiveness of the GIHPIs were grouped into a list of common attributes by 2 reviewers (D.R.B. and N.D.K.). One MCP provided program descriptions that were unclear and could not be interpreted across all behavior and disease categories; these descriptions were excluded from the analysis. Seven descriptions of GIHPIs were excluded because of insufficient data. The reviewers compared their lists and agreed to a final list of attributes. Each reviewer independently assigned selected attributes to each program description and measure of effectiveness, and then the reviewers compared and discussed their final assignments. Kappa statistics were used to measure interrater reliability (range, 0.93–0.99). Second, the number of MCPs reporting each attribute by behavior and disease category was tallied. Descriptive statistics, which included frequencies, proportions, medians, and ranges, were used to analyze all remaining quantitative data. The data set did not include missing values after the exclusions were applied.

## Results

The 20 MCPs varied by number of counties served (range, 1–11; median, 1); number of total patients enrolled (70,000–6,850,484; median, 197,500); number (range, 30,415–1,004,062; median, 141,782) and percentage (range, <1%–100%; median, 93%) of Medi-Cal members enrolled; and number of full-time equivalent health educators employed (range, 1–45; median, 2).

The number of HPIs (n = 194) and GIHPIs (n = 137) delivered by MCPs varied by behavior and disease category (Figure). All MCPs provided healthful eating and physical activity interventions, and only 11 offered CVD management programs or alcohol abuse prevention programs. Eighteen MCPs offered high-impact healthful eating interventions, and only 4 offered high-impact alcohol abuse prevention programs.

**Figure:**
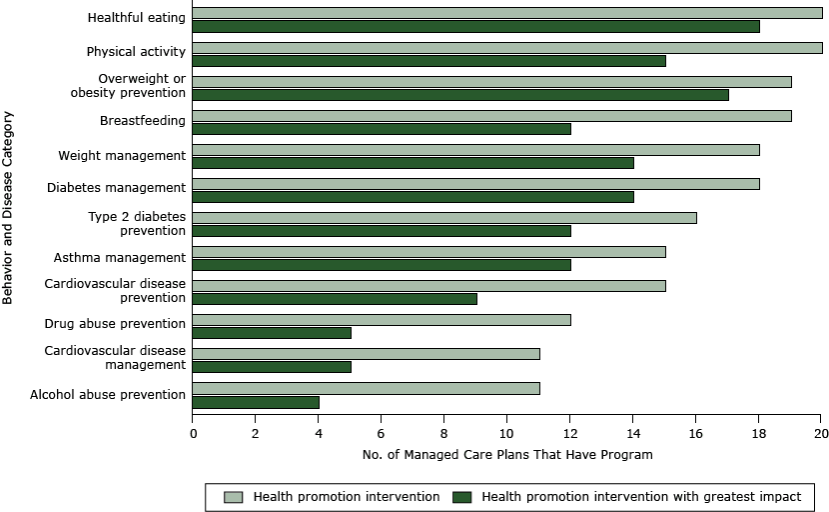
Number of Medi-Cal managed care plans that have a general health promotion intervention or a health promotion intervention with the greatest impact on health, by behavior or disease category, California, 2012. Twenty of 21 managed care plans responded to an online survey. **Table:** 

Behavior or Disease Category	Have a Health Promotion Intervention, n	Have a Health Promotion Intervention With Greatest Impact, n
Healthful eating	20	18
Physical activity	20	15
Overweight or obesity prevention	19	17
Breastfeeding	19	12
Weight management	18	14
Diabetes management	18	14
Type 2 diabetes prevention	16	12
Asthma management	15	12
Cardiovascular disease prevention	15	9
Drug abuse prevention	12	5
Cardiovascular disease management	11	5
Alcohol abuse prevention	11	4
Total	194	137

Educational materials, one-on-one education, and group classes were the 3 most frequently cited delivery methods for GIHPIs (Table 1); 99 of 137 (72%) GIHPIs had 2 or more attributes. 

**Table 1:** Attributes of Health Promotion Interventions With the Greatest Impact, by Behavior and Disease Category, Medi-Cal Managed Care Plans (n = 20), California, 2012^a^^,^^b^

Behavior or Disease Category (No. of Plans That Have an Intervention)	Attribute											
	Educational Materials	One-on-One Education	Group Classes	Referral to Clinical Resources	Health Risk Appraisal and Screening	Disease Management and Self-Management Tools	Incentives for Members	Referral to Community Resources	Health Coaching	Case Management	Resources for Providers	Incentives for Providers
Healthful eating (n = 18)	6	5	9	7	7	2	3	1	2	0	1	0
Physical activity (n = 15)	6	5	3	5	3	2	5	3	4	0	0	0
Alcohol abuse prevention (n = 4)	3	1	1	0	0	0	0	2	0	1	0	0
Drug abuse prevention (n = 5)	3	1	1	0	1	1	0	3	0	0	0	0
Breastfeeding (n = 12)	8	7	1	4	0	1	1	2	0	2	2	0
Overweight/obesity prevention (n = 17)	6	5	10	8	6	2	4	3	3	0	0	0
CVD prevention (n = 9)	3	3	3	2	3	1	1	0	1	2	0	1
Type 2 diabetes prevention (n = 12)	4	6	6	1	4	6	1	0	0	1	0	1
Weight management (n = 14)	5	4	5	7	4	5	3	3	3	0	1	0
CVD management (n = 5)	5	1	2	2	2	0	1	0	1	1	0	0
Diabetes management (n = 14)	6	4	2	4	5	7	2	0	0	1	1	0
Asthma management (n = 12)	3	6	1	1	5	4	1	0	0	5	2	0
**Total**	58	48	44	41	40	31	22	17	14	13	7	2

Abbreviation: CVD, cardiovascular disease.

a Source of data: survey of the lead health educator from 20 of 21 contracted managed care plans in the Medi-Cal system.

b When a health promotion intervention applied to more than one behavior and disease category, it was counted and analyzed as a unique entry. For example, if the same intervention was noted in the category of healthful eating and the category of overweight/obesity prevention, it was analyzed and counted in both categories.

Most GIHPIs were aimed at tertiary prevention (120 of 137 [88%]), followed by primary prevention (44 of 137 [32%]) and secondary prevention (35 of 137 [26%]). The number of minutes or hours of education provided by the interventions varied (range, 15 min–48 h); the median was 2.4 hours. The number of Medi-Cal members who participated in the interventions also varied (range, 0–44,289); the median number of members reached by an intervention was 265.

Of the 137 GIHPIs, 86 (63%) described how effectiveness was measured (Table 2). The 3 most frequently cited measures across all categories were measures of behavior change (eg, changes in dietary intake or physical activity); knowledge; and improved disease management, demonstrated by changes in clinical measures or laboratory values. According to respondents who provided further details about their method of measurement for these 3 measures, 35 of 45 (78%) GIPHIs were evaluated using pre-assessments and post-assessments involving surveys, laboratory tests, or other clinical metrics.

**Table 2:** Measures of Effectiveness Used by Managed Care Plans for Health Promotion Interventions With the Greatest Impact, by Behavior and Disease Category, Medi-Cal Managed Care Plans (n = 20), California, 2012^a^

Behavior or Disease Category^c^	Measure^b^												
	Behavior Change	Knowledge Gain	Improved Disease Management^d^	Patient Satisfaction	Participation	No. of Members Receiving Coaching/ Counseling	Health Care Effectiveness Data and Information Set	Attitudes and Self-Efficacy	Hospital Utilization	Change in Body Mass Index	No. of People Screened	Frequency of Clinical Visits and Access to Clinical Services	No. of Materials Requested or Distributed
Healthful eating (n = 14)	7	5	0	5	1	3	0	1	0	3	0	1	0
Physical activity (n = 9)	5	2	0	4	4	2	0	0	0	1	0	0	0
Alcohol abuse prevention^e^ (n = 1)	0	0	0	0	0	0	0	0	0	0	0	0	0
Drug abuse prevention (n = 2)	0	0	0	0	1	0	0	0	0	0	0	0	1
Breastfeeding (n = 8)	1	1	0	0	2	0	1	1	0	0	0	1	2
Overweight/ obesity prevention (n = 8)	4	2	0	0	3	4	0	3	0	2	1	1	0
CVD prevention (n = 6)	0	2	2	1	0	0	2	1	2	0	1	0	0
Type 2 diabetes prevention (n = 8)	1	2	3	1	1	0	3	1	2	0	1	0	0
Weight management (n = 3)	1	2	0	0	1	2	0	1	0	1	1	1	0
CVD management (n = 3)	0	0	0	1	0	0	0	0	2	0	0	0	0
Diabetes management (n = 12)	1	2	6	2	0	0	3	1	2	0	2	1	1
Asthma management (n = 12)	1	1	5	1	1	0	2	1	1	0	0	0	0
**Total**	21	19	16	15	14	11	11	10	9	7	6	5	4

Abbreviation: CVD, cardiovascular disease.

a Source of data: survey of the lead health educator from 20 of 21 contracted managed care plans in the Medi-Cal system. Not all plans reported using measures of effectiveness.

b The following measures were excluded from the table because of low frequencies: self-reported health status/disease management, medication use/compliance, number of incentives distributed, timeliness of educational material mailings, and educational material comprehension.

c Some managed care plans described multiple measures of effectiveness; therefore, the number of measures may be greater than the number of managed care plans in each behavior or disease category.

d Measured as changes in clinical measures or laboratory values.

e Timeliness of educational material mailings and educational material comprehension were measures of effectiveness for alcohol abuse prevention.

When asked how DHCS could complement the MCPs’ health promotion efforts, 11 of 20 (55%) MCPs suggested that DHCS provide best practice guidelines, share effective interventions, and specify the level of interventions required to meet contractual requirements. In addition, 5 of 20 (25%) MCPs recommended the provision of materials that are culturally, linguistically, and educationally appropriate for Medi-Cal members.

Fourteen or more MCPs reported referring Medi-Cal members to food and nutrition assistance, shelter, utilities, or financial support services (Table 3). Seven or fewer MCPs cited referrals to education, employment, childcare assistance, or the 211 telephone line.

**Table 3:** Medi-Cal Managed Care Plans (n = 20) Referring Medi-Cal Members to Community Assistance Programs, California, 2012^a^

Assistance Program	Number (N = 20)
Special Supplemental Nutrition Program for Women, Infants, and Children (WIC)	18
Food banks	17
Domestic violence shelters	16
CalFresh (Supplemental Nutrition Assistance Program)	16
Housing assistance	15
Homeless shelters	14
Temporary Assistance for Needy Families (TANF)	14
Utilities assistance (eg, electricity, home heating, telephone service)	14
English proficiency programs	7
Job training and placement	6
Childcare assistance	6
Adult education/General Educational Development (GED) test preparation	4
Vocational education programs	4
211 Telephone line	1
Do not refer to community assistance programs	1
No response	1

a Source of data: survey of the lead health educator from 20 of 21 contracted managed care plans in the Medi-Cal system.

Twelve MCPs reported sponsoring physical activity events, such as cycling, walking, and running events. Five sponsored health fairs and food pantries, 4 invested in farmers markets, 2 supported community and school gardens, and 3 did not sponsor community activities.

## Discussion

The delivery of HPIs and GIHPIs across behavior and disease categories varied in our study. The variability of HPIs was surprising, given that each MCP contract calls for the implementation of educational interventions in risk reduction, healthful lifestyle, health condition management, and self-care and also specifies the risk factors and diseases covered by our investigation (8–10). Several factors may explain the differences between contract expectations and practice. First, the MCPs may have emphasized certain behavior and disease topics because of local needs and the special characteristics of the populations served, although these factors do not fully explain the degree of variation found. Second, use of a standardized, valid health risk appraisal has not been required of MCPs, making it difficult to judge risk and tailor intervention delivery accordingly. Third, the lack of a performance monitoring and evaluation system to track HPIs confounds accountability. To advance systemwide health promotion, monitoring and evaluation are essential. It was also surprising that some plans did not report GIHPIs, possibly indicating that these MCPs did not judge any of their interventions to be worthy of greatest impact status.

Multiple attributes were identified for the GIHPIs. Educational materials, one-on-one education, and group classes were the top 3 delivery modes. Although helpful in improving knowledge, these approaches may not address the complex interplay of determinants that shape near- and long-term health behaviors, including self-efficacy, social support, organizational policies, and the environment in which people live (16). Of the 137 GIHPIs, 86 (63%) described how effectiveness was measured; behavior change, knowledge gain, and improved disease management were the most commonly used measures. Most GIHPIs focused on tertiary prevention, median intervention hours were limited, and median Medi-Cal member participation was low. These findings are consistent with those of another assessment of coverage, utilization, and evaluation of health-promoting programs among California’s commercial health plans (17). The study found that most health maintenance organizations (HMOs) used brochures to address health issues and offered free educational classes. Outcome measures used by HMOs to evaluate the impact of their programs included member satisfaction surveys, participation rates, behavior change, and changes in health status. They also found low patient participation rates in health plan–sponsored health promotion programs.

Although health education programs are important, they often have limited impact on health behaviors or long-term health status by themselves. They are more effective when coupled with interventions that address the many determinants of individual and population health (18,19). A growing body of research shows that the health care delivery system’s focus on treating medical conditions typically overshadows and neglects the significant role that social needs — such as food security, safe housing, and employment assistance — play in health, especially among vulnerable populations (18,20). Our study found mixed results in MCP efforts to support or improve selected determinants of health and invest in health-promoting community activities. Most MCPs referred Medi-Cal members to food assistance, shelter, utilities, and financial support services; referrals to education and job-related resources were limited. Most MCPs invested in some type of physical activity event, and a few sponsored health fairs, food pantries, farmers markets, or community and school gardens. Several MCPs did not invest in any community activities. The MCPs could benefit from greater application of the USPSTF guidelines, *The Guide to Community Preventive Services*, and other sources of information on effective HPIs to improve the delivery of whole-person care (11,21).

To our knowledge, ours is the first study to describe the characteristics of HPIs conducted in a state Medicaid program. This work is timely, given that the Affordable Care Act offers opportunities to increase access to preventive services through the expansion of Medicaid and through several provisions in the law that provide incentives to states to increase access to Medicaid- and CHIP-covered preventive services (6). The results of this study are important for population health in California; nearly 10 million Californians are receiving full-scope Medi-Cal services from MCPs. Because federal law and policy determined by the Centers for Medicare and Medicaid Services provide oversight for all Medicaid MCPs, our results suggest areas in which health promotion and disease prevention and management activities could be enhanced in Medicaid plans outside of California.

Our study has several limitations. First, responses to the questions were self-reported and subject to possible comprehension, memory, and other reporting errors. Second, the MCPs are under contract with DHCS; therefore, responses may reflect a social desirability bias. Third, given the exploratory nature of this study, the GIHPIs were identified independently by each MCP without the use of standardized criteria. Fourth, some behavior and disease categories, such as cancer, were excluded from the assessment to maintain a reasonable number of survey questions. Despite these limitations, the overall patterns of practice in health promotion were striking and consistent with a similarly designed study of commercial health plans in California in the late 1990s (17). The results also provide a real-world snapshot of health promotion services provided to a large, well-defined, low-income population.

The findings of this study indicate that substantial and immediate opportunities exist to improve the delivery and effectiveness of health promotion and disease prevention and management services for Medi-Cal members. Improvements are critical, given that populations of low socioeconomic status have higher morbidity and mortality rates than the general population (22). Establishing an evidence-based, measurable, consensus standard of HPIs and leveraging partnerships with state and local health departments with expertise in health promotion may materially improve service delivery among those most in need of sound preventive services. This call to action is consistent with 11 of 20 (55%) MCPs, which recommended that DHCS provide best practice guidelines, share effective interventions, and specify the level of health promotion and disease prevention and management services required to meet contractual agreements.

To advance health promotion in Medi-Cal, achieve outcomes consistent with high-performing systems, such as Kaiser Permanente and the reengineered Veterans Affairs (VA) Health Care System (23–25), and inform the broader dialogue about preventive care improvements in Medicaid, DHCS plans to determine 1) how the MCPs assess health risks among Medi-Cal members and how risk-related data are used to inform intervention delivery; 2) the best approach to set quality improvement targets and accountability systems, starting with the leading causes of preventable mortality and illness, to ensure that evidence-based interventions are delivered to Medi-Cal members in a timely, prudent, and effective manner; 3) methods to optimize the delivery of the USPSTF A and B recommendations and other evidence-informed best practice interventions (11); 4) opportunities to ensure that health care and community prevention efforts are available, integrated, mutually reinforcing, and address multiple determinants of health; and 5) methods to implement a monitoring system for tracking the delivery and performance of HPIs. Such a system could help decision makers deploy resources to the most effective programs while curtailing ineffective programs.

Studies are needed to learn how methods associated with the consistent delivery of preventive services in highly organized delivery systems (eg, the VA Health Care System, Kaiser Permanente, Group Health Cooperative) can be applied more effectively in Medicaid health plans, which typically contract with a diverse network that might include federally qualified health centers, independent practice associations, large group practices, and specialty groups. On a broader scale, to achieve the prevention and health promotion and disease prevention and management targets outlined in the National Strategy for Quality Improvement in Health Care and the National Prevention Strategy, additional applied research is needed to understand how to effect systemwide changes that advance population health in Medicaid (26,27).
